# Clinical Gleanings

**Published:** 1895-07

**Authors:** 


### CLINICAL GLEANINGS.

THE internal use of sulphur is again strongly urged in diseases of the skin of animals, and is considered by some sheep-raisers of Cape Colony more effective in these animals and less dangerous every way than the sheep-dips in general use. It is suggested that it may be given mixed with the usual allowance of salt when required.

THE use of salicylicate of soda and acetanilid in half-drachm doses each, given every two to four hours, has proven very effective in the treatment of rheumatism and rheumatoid influenza at the Philadelphia Veterinary Sanitarium. In those of a muscular character the results have been quick and radical, while those which were articular in character were less rapid, and with one exception finally yielded.

